# Tracing the pathways to Yathrib (Al-Madinah): A GIS-based analysis of settlement patterns and ancient trade networks

**DOI:** 10.1371/journal.pone.0348677

**Published:** 2026-05-07

**Authors:** Mohamed Metwaly, Abdullah Alshami

**Affiliations:** Archaeology Department, College of Tourism and Archaeology, P.O. Box, Riyadh, King Saud University, Saudi Arabia; Sathyambama Institute of Science and Technology: Sathyabama Institute of Science and Technology (Deemed to be University), INDIA

## Abstract

Ancient human activities in the arid Arabia peninsula are considered one of interesting research subjects in the landscape archaeological field to understand the relationships between human occupation strategies, material culture, and the natural environment in the past. Focusing on Al-Madinah Region of the Arabian Peninsula, a landscape of profound historical and archaeological significance, the research integrates Multi-Criteria Spatial Analysis (MCSA) using Geographic Information Systems (GIS) and the Analytic Hierarchy Process (AHP) with Least-Cost Path (LCP) modeling. The study aimed to (i) evaluate environmental suitability for historical settlements and (ii) reconstruct optimal caravan routes converging on Yathrib (Al-Madinah). Six principal environmental and topographic variables were evaluated: lithology, elevation, slope, drainage density, distance to watercourses (wadis), and the soil classification. A balanced weighting system was implemented (Consistency Ratio = 0.01), where proximity to watercourses, slope, and soil were identified as the primary controlling factors. The resulting settlement suitability map identified 32.01% of the study area as highly suitable. For the reconstruction of caravan routes, a cost surface was generated based on four parameters: distance to watercourses, slope, elevation, and lithology to simulate LCPs to major archaeological sites, including Khaybar, Al-Hinakiyah, Dadan–AlUla–Hegra, and the coastal port of Al-Jar. Quantitative validation of these modeled routes against verified historical trade stations demonstrates that hydrological accessibility and terrain favorability jointly governed patterns of ancient human settlement and mobility, offering a replicable methodology for exploring trade networks and habitation dynamics in arid landscapes.

## 1. Introduction

Understanding the spatial suitability of ancient human settlements in arid environments is fundamental to landscape archaeology, as environmental constraints strongly influence patterns of habitation, mobility, and cultural interaction. The Al-Madinah region, located in the western Arabian Peninsula, presents a particularly significant case study. Archaeological and historical evidence indicates continuous human occupation from the Early Stone Age through the Islamic period to the present day [1,2]. Despite extreme aridity, limited surface water availability, and complex topographic variability, ancient communities successfully adapted to these environmental constraints, developing settlement systems closely linked to resources accessibility and landscape affordances. These adaptations not only enabled sustained habitations but also contributed to the emergence of Al-Madinah as a major regional center, particularly during the pre-Islamic and early Islamic periods.

Al-Madinah’s strategic geographic location positioned it as a key node within extensive trade and pilgrimage networks connecting southern Arabia with the Levant and Mediterranean regions [2–4]. These caravan routes facilitated the transport of valuable commodities such as frankincense, myrrh, spices, and textiles across vast desert landscapes. The domestication of the Arabian camel during the late second millennium BCE marked a critical technological innovation that enhanced long-distance mobility by allowing caravans to traverse arid terrain with minimal water dependence. As a result, caravan routes evolved into structured networks of interconnected pathways, linking resource zones, settlements, and logistical nodes such as water sources and caravan stations. These networks played a central role in shaping settlement distribution and regional socio-economic connectivity throughout Arabia [2–4].

Despite substantial historical and archaeological documentation of Arabian trade systems, several key questions remain unresolved regarding the spatial logic underlying route selection and settlement placement. In particular, the relative influence of environmental variables including topography, hydrology, and terrain suitability on the configuration of ancient movement corridors and associated settlements remains insufficiently quantified. Addressing these questions requires analytical approaches capable of integrating geoenvironmental datasets with spatial modeling techniques to reconstruct and evaluate potential movement pathways.

Recent advances in Remote Sensing (RS) and Geographic Information Systems (GIS) have significantly enhanced the ability to analyze archaeological landscapes, particularly in arid regions where surface preservation of ancient features is often exceptional [5–9]. High-resolution satellite imagery, digital elevation models, and geospatial analysis tools enable the identification of terrain characteristics and environmental constraints influencing human activities and movements. Among these approaches, Least-Cost Path (LCP) analysis has emerged as a widely adopted method for simulating human mobility across heterogeneous landscapes. By modeling movement across cost surfaces derived from environmental variables such as slope, proximity to water, soil classification, and terrain characteristics, LCP analysis provides a quantitative framework for reconstructing plausible ancient routes and evaluating their environmental feasibility [10–12].

Globally, LCP modeling has been successfully applied to reconstruct ancient communication networks, including major transregional systems such as the Silk Road and other caravan routes, demonstrating the critical influence of environmental and ecological factors on route development [12,13]. In the Arabian Peninsula, similar approaches have contributed to understanding the general configuration of the Incense Route and its associated corridors [14]. However, many previous studies have focused primarily on regional-scale reconstructions or historical interpretations, with limited integration of systematic multi-criteria spatial evaluation and quantitative weighting of environmental variables at the local landscape scale [2,4,15].

Furthermore, conventional LCP approaches often rely on generalized assumptions regarding movement efficiency and may not adequately account for the relative importance of specific environmental factors in shaping localized mobility patterns and settlement connectivity [12,16]. This limitation is especially significant in arid environments, where access to ephemeral water sources such as wadis, alongside land capability and topographic ease, likely played a decisive role in determining both settlement locations and movement corridors.

Accordingly, a critical research gap exists in quantitatively evaluating how multiple environmental and topographic variables, collectively influenced the spatial configuration of ancient trade corridors and settlement networks in the immediate hinterland of Al-Madinah. While previous research has demonstrated the general applicability of GIS and LCP modeling in Arabian contexts, few studies have integrated a structured Multi-Criteria Evaluation (MCE) framework using the Analytic Hierarchy Process (AHP) to systematically quantify environmental suitability and test its relationship with reconstructed mobility pathways at a high spatial resolution [7–9]. To address this gap, the present study integrates AHP-based multi-criteria spatial evaluation with Least-Cost Path analysis to model environmentally optimal movement corridors and assess their relationship with settlement suitability in the Al-Madinah region. Specifically, this research tests the hypothesis that synergistic interaction between hydrological features, terrain, soil types, and geological factors, exerted a dominant influence on the spatial organization of ancient mobility and settlement systems. By combining remote sensing datasets, GIS-based environmental modeling, and quantitative spatial analysis, the study provides new insights into the environmental determinants of human mobility and contributes to a more refined understanding of settlement dynamics and landscape connectivity in arid archaeological contexts.

## 2. The study area

**Fig 1:**
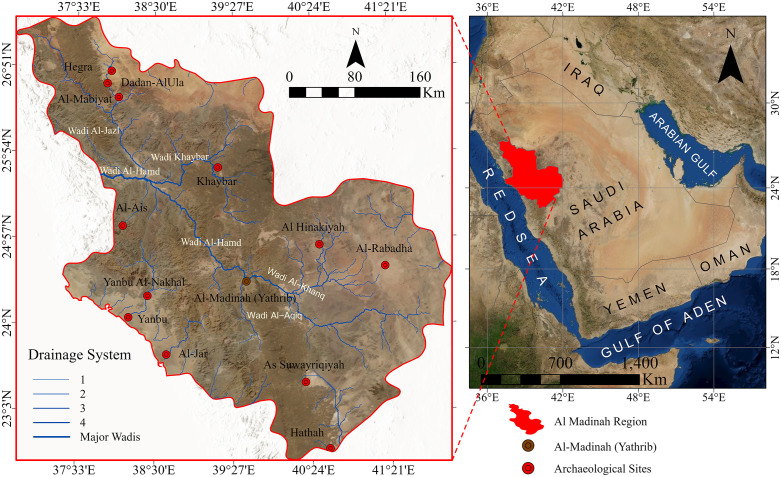
Location map of the Al-Madinah region illustrates current administrative boundaries, principal archaeological sites (notably ancient Yathrib), and main wadis (Maps created in May 2025 using ArcGIS Pro (Esri, Inc., 2023).

The study area is situated in the northwestern part of the Arabian Peninsula, within the administrative boundaries of Al-Madinah Al-Munawarah, in the western region of the Kingdom of Saudi Arabia. It is bordered by the regions of Tabuk to the north, Makkah to the south, Hail, Qassim, and Riyadh to the east, and the Red Sea to the west (Fig. 1). This strategic geographic positioning historically established Al-Madinah as a central hub along the major ancient trade and pilgrimage routes that linked southern Arabia with the Levant and the Mediterranean basin.

Al-Madinah is among the most historically and culturally significant urban centers in the Arabian Peninsula, with continuous settlement evidence dated back several millennia [3,15]. The region experiences a hot, arid desert climate, marked by extremely high summer temperatures, mild winters, and sparse, irregular rainfall. These climatic constraints have long influenced patterns of habitation, agriculture, and water management strategies, shaping the spatial distribution of ancient and modern settlements [17].

Geographically, the city of Al-Madinah lies approximately 480 kilometers north of Makkah, positioned within a fertile oasis that historically sustained human occupation and agriculture activities. The area occupies a natural basin framed by two extensive volcanic lava fields; Harrat Waqim to the east and Harrat al-Wabrah to the west. These geomorphological features, characterized by rugged basaltic terrain, acted as significant natural barriers influencing movement and settlement. This setting, interlaced by a network of wadis and seasonal drainage channels, and characterized by specific soil compositions such as Calciorthids in the alluvial plains, provided access to surface and groundwater resources essential for sustaining settlement continuity over time.

The city’s hydrological and ecological diversity, combined with its strategic location along the overland incense and trade routes connecting southern Arabia to the Levant (particularly Gaza), made it an indispensable waypoint for caravans and pilgrims [2]. These natural and cultural factors together enhanced Al-Madinah’s role as both a center of agricultural productivity and a logistical crossroads throughout antiquity and the early Islamic period.

In the context of this study, the unique environmental, hydrological, and topographic conditions of Al-Madinah make it a pivotal region for spatial analysis. Its landscape provides a valuable case study for evaluating ancient settlement suitability and modeling least-cost pathways of trade and pilgrimage routes across northwestern Arabia [3,18].

## 3. Geological and environmental setting

Al-Madinah region exhibits a geologically diverse and environmentally dynamic framework that has profoundly shaped patterns of human settlement, mobility, and land-use over millennia. Laying along the western margin of the Arabian Shield, the region encompasses a complex assemblage of igneous, metamorphic, and sedimentary rocks, interspersed with extensive ancient volcanic fields (Harrats) that dominate much of the surface landscape [19]. This lithological diversity and old volcanic intensity have been pivotal in determining both the spatial distribution of settlements and the feasibility of ancient movement corridors across the region.

Among these features, Harrat Rahat stands as the most prominent volcanic field, extending across the southern and southeastern sectors of Al-Madinah and partially encircling its urban and agricultural areas. Composed primarily of Tertiary to Quaternary basaltic flows, Harrat Rahat forms a rugged, uneven terrain characterized by high thermal inertia, low permeability, and limited soil development. These properties historically constrained water infiltration and storage, influencing the localization of oases, wells, and habitation zones within the adjacent lowlands.

**Fig 2:**
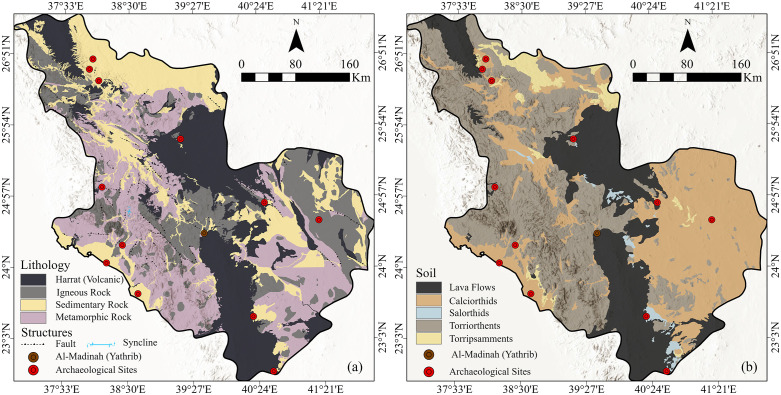
(a) Lithological map and (b) soil classification map of Al-Madinah Region. The geological and pedological data were digitized and spatialized using ArcGIS Pro. 2023, based on the geological maps provided by the Saudi Geological Survey (SGS) [23].

To the northeast lies part of Harrat Khaybar, one of the largest volcanic features in the Arabian Peninsula, known for its complex geomorphology, including overlapping basaltic flows, pyroclastic deposits, and numerous volcanic cones [19,20]. Together, these volcanic systems form a network of natural topographic barriers that historically channeled ancient caravan routes and constrained settlement expansion into well-watered inter-harrat basins and wadis (Fig 2a).

The soils of the Al-Madinah region reflect this geological variability by the lateral distribution of the soil along the low land areas and between surface basement and volcanic features (Fig 2b). These soils are predominantly classified as Aridisols and Entisols with specific taxonomic groups including Typic Calciorthids and Typic Torriorthents dominating the fertile alluvial plains and wadi basins [17]. These soil types differ significantly in terms of texture, fertility, and water storage capacity, directly influencing ancient agricultural potential and sustainable land use. For instance, the Calciorthids, characterized by calcium carbonate accumulation, provided stable and fertile subsurface for ancient palm groves and permanent settlement around the Al-Madinah oasis. Whereas saline and coarse-textured soils found in peripheral volcanic zones limited long-term occupation.

Collectively, the geological and environmental complexity of Al-Madinah not only constitutes the physical foundation of historical settlements but also serves as a critical interpretive framework for reconstructing ancient land-use strategies, mobility networks, and trade route dynamics across northwestern Arabia. The integration of these natural parameters is therefore essential for understanding the spatial logic of settlement suitability and the environmentally conditioned pathways that linked Al-Madinah to broader trans-Arabian trade systems.

## 4. Topographic characteristics

The Al-Madinah Region displays pronounced topographic diversity, reflecting its complex geomorphological evolution and the cumulative influence of tectonic activity, volcanism, and long-term weathering processes [19]. Situated along the transitional belt between the volcanic highlands of the Hijaz and the inland desert plains of central Arabia, the region forms a distinctive physiographic mosaic that has historically guided patterns of human occupation and mobility.

**Fig 3:**
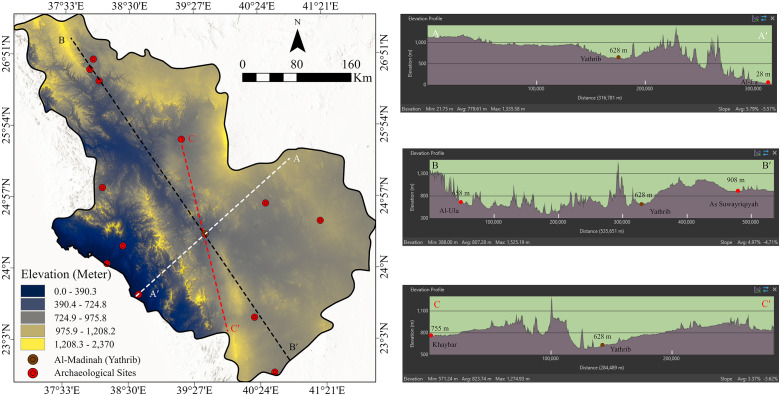
Digital Elevation Model (DEM) of Al-Madinah region. The map illustrates elevation distribution and three strategic profiles (A–A′, B–B′, and C–C′). Topographic data derived from ALOS PALSAR (Maps created in May 2025 using ArcGIS Pro (Esri, Inc., 2023).

Examination of the geological and digital elevation models (Figs 2 and 3) reveals that high-elevation zones correspond primarily to basement complexes and volcanic harrats, while low-lying sectors coincide with fertile alluvial valleys (wadis). Within this landscape lies the oasis basin of Al-Madinah, sustained historically by shallow groundwater and natural springs. This oasis environment provided both agricultural viability and settlement continuity, serving as suitable historical area within an otherwise arid terrain. Moreover, most of the ancient settlement sites along the study area are laying in the local topographic basins. This evidence supported the smart choice of settlement sites and the suitable pass of the trade routes.

Topographically, the region’s structure defines distinct morphological gradients: to the east, the land gently descends into sandy desert plains extending toward the Najd Plateau; to the west, the terrain rises sharply into the Hijaz Mountains, which forms a rugged barrier separating the interior from the Red Sea coastal plain (Fig 3). These natural gradients played a strategic role in shaping ancient mobility systems, particularly the trade and pilgrimage routes by directing movement through accessible corridors and around volcanic or mountainous obstacles.

The city of Yathrib (ancient Al-Madinah) occupies a naturally favorable depression within this system, functioning as a logistical stopover with reliable water resources and cultivable land amid the surrounding volcanic and desert landscapes. To further elucidate the region’s topographic structure, three cross-sections were derived from the DEM dataset (Fig 3):

- Profile A–A′ extends approximately 316.8 km, tracing the transect from the coastal lowlands near Al-Jar in the west to the eastern volcanic highlands. The profile exhibits a gradual ascent toward the central uplands, punctuated by successive ridges and wadies, before descending to Al-Jar, which lies at ~28m above sea level. Al-Madinah basin, centrally positioned at ~628 m, marks an intermediate plateau between the interior and coastal zones.
- Profile B–B′stretching 535.7 km from Al-Ula in the northwest to As Suwayriqiyah in the southeast, displays a markedly rugged topography. Al-Ula begins at ~658 m, rising to a maximum of ~1,525 m across steep escarpments and mountainous sectors before declining to ~908 m near As Suwayriqiyah. This transect highlights the pronounced relief and structural barriers that constrained ancient movement across the northwestern corridor.
- Profile C–C′, linking Khaybar to Yathrib over ~284.5 km, shows a relatively moderate relief compared to the other transects. Elevation declines gradually from ~755 m at Khaybar to ~628 m at Yathrib, with gentle undulations along the route. The smoother topography of this sector likely offered a more accessible travel corridor, consistent with historical records of movement and settlement between these centers.

Collectively, these elevation profiles underscore the strategic topographic positioning of Yathrib—protected by volcanic fields yet accessible through multiple natural corridors. This advantageous setting facilitated its emergence as both a permanent settlement center and a nodal waypoint in the ancient trade and pilgrimage networks that traversed western Arabia.

## 5. History of Al-Madinah area

The historical trajectory of the Al-Madinah region, formerly known as Yathrib, forms an integral part of the broader cultural and geopolitical history of northwestern Arabia. Strategically positioned at the crossroads of major caravan routes linking southern Arabia with the Levant, Yathrib functioned as both a fertile oasis and a key exchange center within long-distance trade networks. Its advantageous environmental attributes fertile soils, fresh groundwater, and proximity to volcanic highlands fostered continuous human occupation, economic interaction, and cultural convergence over several millennia.

The city’s significance long predates the rise of Islam. References to Yathrib appear across multiple epigraphic, historical, and classical geographical sources, attesting to its early integration within the trade and political systems of ancient Arabia. Textual and historical evidence indicates that Yathrib was an established and regionally significant settlement from at least the first millennium BCE onward. The earliest reference appears in Babylonian sources, where King Nabonidus in the 6th century BCE recorded Yathrib among the territories under his influence, highlighting its early political and strategic importance. In subsequent centuries, inscriptions and historical records from the Minaean and later Nabataean civilizations further attest to Yathrib’s active participation in long-distance trade and diplomatic networks across Arabia. This regional prominence continued into the Himyarite period, reflecting sustained occupation and integration within south Arabian commercial systems. By the Greco-Roman era, Yathrib was sufficiently well known to be documented by Claudius Ptolemy in the 2nd century CE, who referred to the settlement as “Lathrippa,” confirming its recognition within classical geographic and cartographic traditions [2–4,15,18].

Archaeological evidence, including rock art, lithic scatters, and settlement remains, indicates continuous settlements in the wider Al-Madinah region since prehistoric times. Despite its longevity, pre-Islamic Yathrib appears to have maintained a decentralized socio-political structure, characterized by tribal affiliations and localized authority rather than a formalized state apparatus. Scholars agree that no evidence exists for centralized governance or royal institutions prior to the 7th century CE, when the city became the center of the emerging Islamic polity [18].

Yathrib’s dual role as a caravan station and agricultural oasis was sustained by its hydrological system, driven by seasonal wadies and groundwater-fed basins. These natural systems supported both subsistence agriculture and commercial exchange, transforming the oasis into a cosmopolitan settlement that attracted diverse populations. The city’s participation in trans-Arabian trade linked it economically and culturally to southern Arabian polities, northern groups such as the Nabataeans and Ghassanids, and the broader Incense Route that connected Arabia to the Mediterranean [2,4].

**Fig 4:**
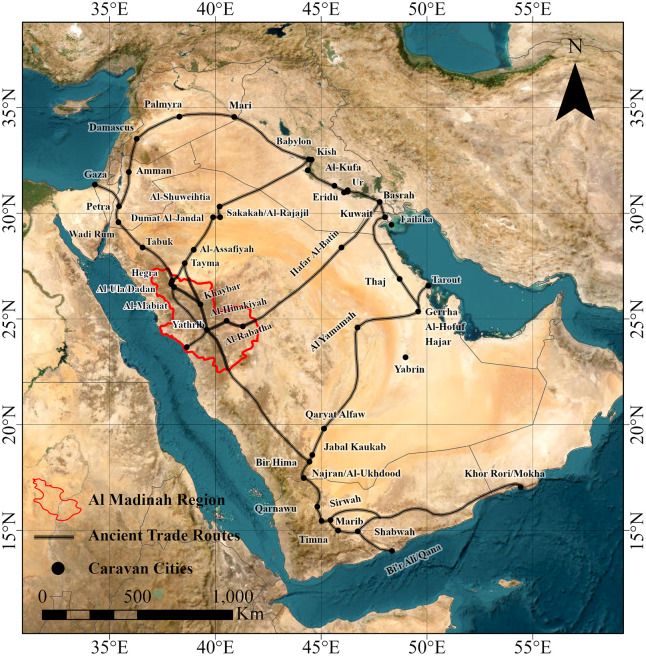
A map illustrating the major ancient caravan trade routes in the Arabian Peninsula (Maps created in May 2025 using ArcGIS Pro (Esri, Inc., 2023) and modified from [1].

This historical significance is further contextualized by the spatial configuration of regional settlement center historically linked to Yathrib via overland and coastal caravan routes (Fig 4). To the north, the archaeological complex of Al-Ula and Dadan represents one of the earliest urban and trade centers of Arabia. Along the western coastal plain, the ports of Al-Jar and Yanbu functioned as maritime extensions of inland caravan routes. To the northwest, Al-Ais served as a strategic intermediate stopover, while to the east, sites such as Al-Rabadha and Al-Hinakiyah lay along interior travel corridors. Further south, Hathah and As-Suwayriqiyah marked additional centers within the network of pilgrimage and trade routes [1,2,21].

Although these sites vary in chronology, function, and archaeological character, their spatial distribution delineates the broader regional networks that structured Yathrib’s accessibility and economic vitality. Within the framework of the present study, their inclusion in least-cost path (LCP) modeling serves to reconstruct the environmental and cultural parameters that governed ancient mobility and interaction across the northwestern Arabian landscape.

## 6. Datasets and methods

The study employed an integrated geospatial and remote-sensing framework to evaluate ancient settlement suitability across the Al-Madinah region and to reconstruct historical least-cost pathways (LCPs) converging on Yathrib (ancient Al-Madinah). The methodological approach combined digital elevation modeling (DEM), soil and geological mapping, and spatial multi-criteria decision analysis (MCDA) within a GIS environment. By integrating these parameters, quantitative reconstruction of environmental affordances influencing habitation and mobility in northwestern Arabia. To ensure methodological rigor and avoid circular reasoning, the settlement suitability model and the LCP cost surface were developed as distinct analytical components, validated against known archaeological and historical stations.

### 6.1 Data sources and preprocessing

The analysis was primarily based on high-resolution and regionally calibrated datasets. Terrain parameters including elevation, slope, drainage density, and distance to water networks were derived from Advanced Land Observing Satellite (ALOS) PALSAR DEMs at 12.5 m spatial resolution [22]. These data were resampled and hydrologically corrected to ensure accurate topographic representation and to remove potential sink anomalies prior to slope and flow accumulation analysis.

Geological and lithological datasets were obtained from [23], providing the spatial distribution of bedrock units, volcanic fields (Harrats), and soil classifications. These data sets were essential for evaluating substrate stability, land capability, and resource availability. Specifically, soil types predominantly Aridisols and Entisols (including Typic Calciorthids and Typic Torriorthents) were utilized as a stable proxy for ancient agricultural viability and land-use potential, replacing modern multispectral indices to ensure chronological consistency with the archaeological sites and features along the study area.

**Fig 5:**
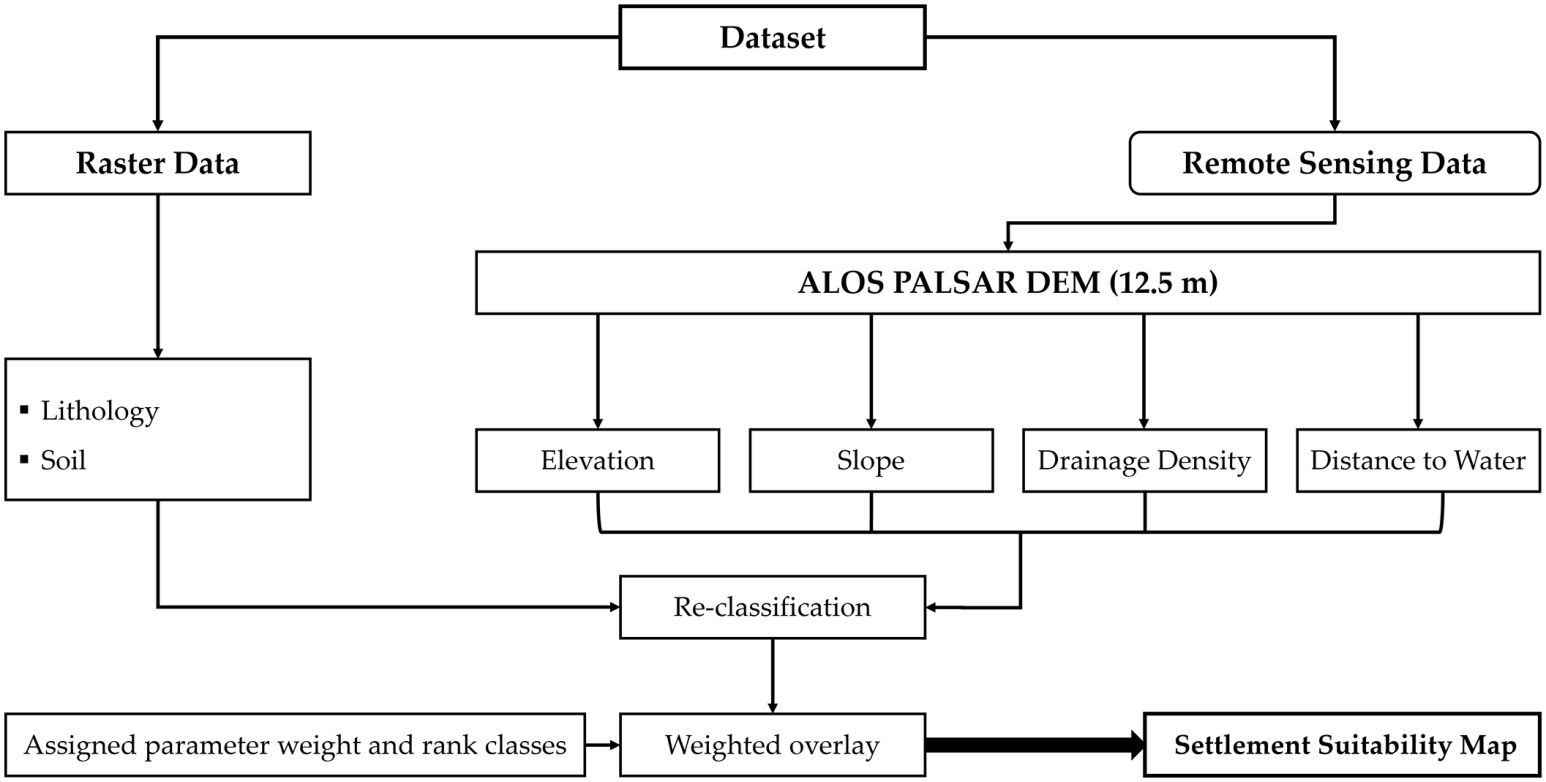
Workflow illustrates the data sources, processing steps, and analytical procedures used to generate the settlement suitability map for the Al-Madinah region.

All spatial layers underwent reprojection, and normalization to a unified coordinate system (WGS 1984 UTM Zone 37N). Following the methodological workflow (Fig 5), the processed layers were then classified into suitability categories based on their inferred influence on settlement potential (e.g., low slope and fertile soil types indicating higher suitability), ensuring analytical consistency across all input variables.

### 6.2 Analytical Hierarchy Process (AHP) and weighted overlay

To quantitatively evaluate the relative influence of environmental parameters on settlement suitability, the Analytic Hierarchy Process (AHP) [24] was employed as a structured multi-criteria decision-making framework. Pairwise comparisons were conducted using an iterative empirical approach to derive normalized weights for each environmental criterion, including slope, proximity to water, soil type, and lithology. Weight distributions were validated against the spatial density of documented archaeological sites within the study area to ensure objective environmental representation. To achieve a balanced model and eliminate potential bias, equal weights (25% each) were assigned to the factors; distance to water, slope, and soil. The consistency ratio (CR) of the pairwise comparison matrix was 0.01, indicating a perfect level of internal consistency and ensuring that the resulting factor weights accurately reflect the landscape’s physical reality.

Subsequently, the standardized and weighted spatial layers were integrated using a weighted overlay analysis in ArcGIS Pro (v3.3), [25] to generate a composite settlement suitability map for the administrative extent of Al-Madinah region (Fig 5). This integrated model produced a continuous spatial representation of environmental and topographic factors. The resulting map provides a quantitative basis for identifying areas that offered favorable conditions for past human occupation and mobility (Table 1).

**Table 1:** Matrix of pairwise comparisons for settlement suitability.

Parameter	Distance to Water	Slope	Soil	Elevation	Drainage Density	Lithology	Priority Vector – PV	normalized principal Eigenvector	Weight %
**Distance to Water**	1	1	1	2	4	4	0.25	25.00	25
**Slope**	1	1	1	2	4	4	0.25	25.00	25
**Soil**	1	1	1	2	4	4	0.25	25.00	25
**Elevation**	0.5	0.5	0.5	1	2	2	0.125	12.50	13
**Drainage Density**	0.25	0.25	0.25	0.5	1	1	0.0625	6.25	6
**Lithology**	0.25	0.25	0.25	0.5	1	1	0.0625	6.25	6
**Sum**	4	4	4	8	16	16	1	100.00	100
CR = 0.01									

### 6.3 Least-Cost Path (LCP) modeling

**Fig 6:**
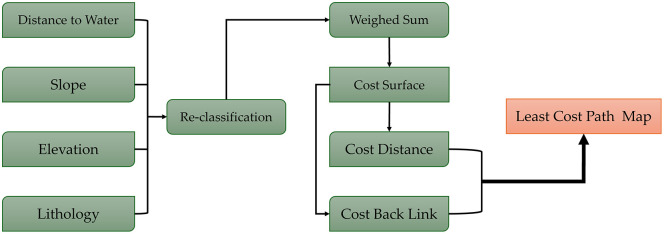
Workflow delineates the construction of the independent cost surface and the subsequent Least-Cost Path (LCP) analysis used to reconstruct ancient mobility and caravan routes.

In the subsequent modeling phase, a dedicated cost surface was developed to reconstruct ancient mobility patterns. This framework assumes that landscape variables, including hydrological proximity, slope, and the geological constraints, directly influenced the movement friction for ancient caravan dynamic. Within this model, wadi systems were characterized as optimal low-resistance corridors, while slope and elevation were reclassified to prioritize minimal energy expenditure. Furthermore, geological ruggedness was integrated by assigning the lowest cost paths are coincident with the sedimentary terrains and the highest resistance related to volcanic fields (Harrat). Using Cost Distance and Cost Backlink functions, a cost-distance matrix was generated, from which optimal movement corridors were computed between Yathrib and surrounding archaeological sites, including Al-Ula, Al-Jar, Khaybar, and Al-Rabadha (Fig 6). This analytical framework effectively distinguishes travel resistance from settlement suitability, allowing for the high-fidelity delineation of probable ancient trade and pilgrimage routes across the northwestern Arabian Peninsula.

### 6.4 Digital Elevation Model (DEM)

**Fig 7:**
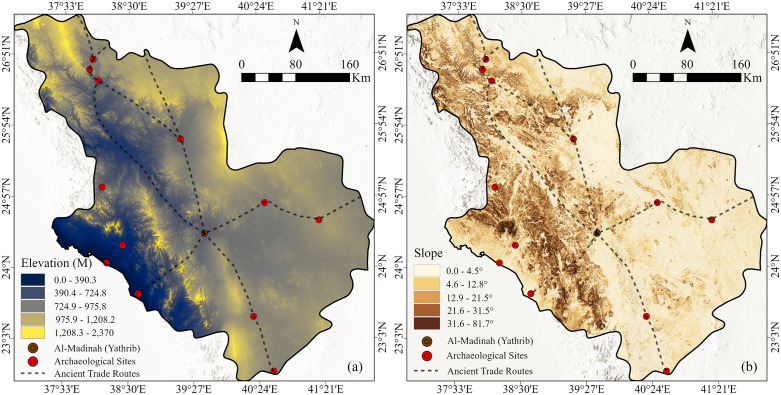
(a) Elevation map and (b) slope map of the study area. Both maps were derived from the ALOS PALSAR DEM data (referenced in Fig 3) (Maps created in May 2025 using ArcGIS Pro (Esri, Inc., 2023).

The Digital Elevation Model (DEM) provides a detailed representation of the topographic variability across Al-Madinah region, reflecting both its complex geological structure and paleogeographic evolution (Fig 7a). Elevations within the study area range from near sea level (0m) along the western coastal plain to approximately 2,370 m across the volcanic highlands (Harrats). This broad altitudinal range emphasizes the region’s geomorphological diversity, shaped by tectonic uplift, volcanic activity, and long-term erosional processes.

A distinct westward elevation gradient is observed toward the Red Sea coast, forming a gradual descent from the elevated basaltic plateaus of the Hijaz mountains. In contrast, the western and north-western sectors display rugged terrain characterized by sharp relief, steep escarpments, and deeply incised valleys, particularly within the Harrat Rahat and Harrat Khaybar systems. Conversely, the lower-elevation zones correspond to expansive alluvial plains and wadi systems, which historically offered greater accessibility, arable soils, and proximity to water sources, thereby fostering ancient human settlement and mobility.

This pronounced topographic heterogeneity played a decisive role in shaping ancient habitation patterns and movement corridors. While elevated zones acted as natural barriers restricting movement, the intervening lowland corridors and wadi basins likely served as preferred travel routes for trade caravans and seasonal migrations. In the context of least-cost path modeling, these lower elevation corridors represent optimal pathways toward Yathrib (Al-Madinah), where accessibility and reduced terrain resistance would have facilitated sustained connectivity between inland settlements and coastal trade centers.

### 6.5 Slope

The slope map (Fig 7b) of Al Madinah region illustrates the spatial variability of surface inclinations, which range from 0.0° to 81.7°. A general east–west gradient is evident, with gentle slopes dominating the eastern parts and progressively steeper gradients occurring toward the west. Flat to very gentle slopes (0–4.5°) are prevalent across the eastern and northeastern sectors, representing relatively stable and low-relief terrain suitable for settlement, agricultural practices, and water accumulation.

Conversely, moderate to steep slopes (12.9–31.5°) are concentrated in the western portions of the study area, reflecting rugged topography that would have posed significant challenges to ancient human occupation and mobility. The very steep zones (greater than 31.6°) mark the most topographically constrained areas, characterized by high surface runoff potential and an increased likelihood of slope instability or landslides conditions generally unfavorable for habitation or sustained activity.

Overall, slope represents a fundamental parameter in historical route reconstruction and landscape suitability analyses. Areas with minimal gradients would have been strategically advantageous for facilitating movement and transport with reduced energy expenditure, particularly within arid and mountainous environments where both terrain and resources imposed natural constraints.

### 6.6 Lithology

Lithological characteristics were considered a key parameter in assessing settlement suitability across Al-Madinah region. The area can generally be categorized into four principal lithological units: harrat (volcanic fields), igneous, metamorphic, and sedimentary formations (Fig 2a). Each unit was evaluated and ranked according to its relative suitability for human habitation, taking into account factors such as terrain stability, permeability, and the availability of materials suitable for ancient construction activities.

Analysis of the spatial distribution of ancient and historical archaeological sites across Al-Madinah area reveals a strong correlation between settlement locations and sedimentary deposits, which are favorable for surface and groundwater accumulation and storage (Fig 2a). In contrast, ancient occupation within the harrat zones appears to have been concentrated around areas of rainwater collection, particularly within volcanic cones and natural basins. Furthermore, examination of the ancient trade routes (Fig 4) demonstrates a distinct preference for pathways traversing sedimentary terrains rather than igneous landscapes. This pattern likely reflects the relative ease of movement over softer surface sedimentary layers, as well as the higher potential for groundwater availability compared to the more resistant and impermeable igneous rocks.

### 6.7 Soil

Soil characteristics were incorporated as a significant environmental determinant of settlement suitability, with data categorized into five dominant types across the region (Fig 2b). Each soil unit was ranked based on its physical properties, including fertility, structural stability, and drainage capacity. Calciorthids were assigned the highest suitability (Rank 5), as they represent the most favorable conditions for ancient settlement due to their moderate fertility and profile stability in arid environments. Conversely, Lava flows were designated the lowest suitability (Rank 1), characterized by their hardened surfaces and negligible water infiltration, which presented substantial constraints to both agricultural potential and ancient infrastructural development.

### 6.8 Drainage density

**Fig 8:**
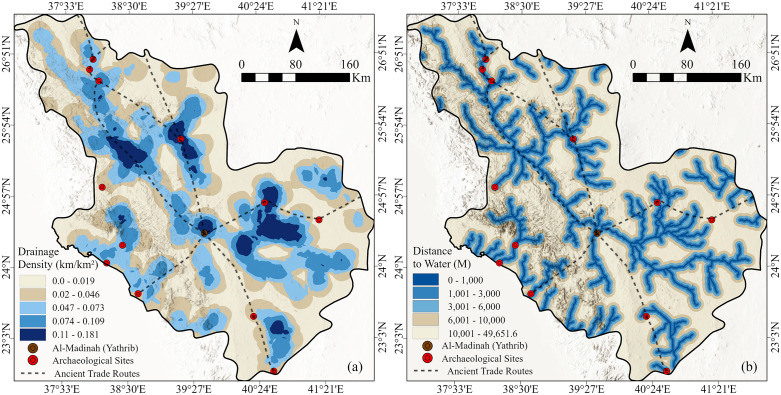
(a) Drainage density map and (b) distance-to-water map of the study area. Hydrological analysis and spatial modeling. These maps are derived from the ALOS PALSAR DEM (12.5m resolution) referenced in Fig 3, (Maps created in May 2025 using ArcGIS Pro (Esri, Inc., 2023).

Drainage density represents a fundamental hydrological parameter that reflects the degree of landscape dissection and the efficiency of surface runoff concentration [26]. It shows the total length of drainage channels (such as rivers, streams, and wadis) per unit area, typically expressed in kilometers per square kilometer (km/km²). Within Al-Madinah Region, this variable is particularly significant for evaluating potential ancient settlement zones, given the region’s arid climate and confidence on ephemeral water sources. The drainage density map (Fig 8a), derived from high-resolution ALOS PALSAR digital elevation data, reveals pronounced spatial variability across the study area. Drainage density is calculated by dividing the total length of all drainage channels features by the area itself. High drainage density values indicate closely spaced channels, often associated with impermeable surfaces, steep slopes, or high runoff potential. While low drainage density reflects fewer channels and is commonly linked to permeable soils, gentle slopes, and greater water infiltration [26].

The geological framework of Al-Madinah, dominated by igneous and metamorphic lithologies, exhibits generally low permeability. Consequently, these rock types restrict groundwater infiltration and storage, emphasizing the importance of surface drainage systems in both runoff collection and limited subsurface recharge. Areas characterized by moderate to low drainage densities serve as key zones for rainwater concentration and groundwater recharge, particularly where wadies intersect permeable alluvial deposits. Such zones are hydrologically advantageous and thus more favorable for ancient settlement, offering intermittent water availability in an otherwise arid environment. Furthermore, analysis of ancient trade routes indicates that many pathways traversed or closely followed regions of moderate drainage density, likely to exploit the enhanced groundwater potential within sedimentary deposits (Fig 8a). This correlation underscores the strategic importance of hydrological factors in shaping historical patterns of movement, settlement, and resource utilization across Al-Madinah landscape.

### 6.9 Distance to water

A Distance to Water map is a spatial representation that quantifies the Euclidean or cost-based distance from any location within a study area to the nearest water source, such as rivers, streams, wadies, springs, or other hydrological features. The map can be achieved by calculating the shortest distance between each raster cell and the nearest mapped water feature, resulting in a continuous surface that reflects spatial variation in water accessibility. The output is typically expressed in meters or kilometers and classified into distance zones [26]. In arid environments such as Al-Madinah Region, where rainfall constitutes the sole source of water, proximity to hydrological features is a critical factor influencing settlement viability. Due to the absence of permanent surface water bodies, transient drainage systems (wadis) function as the principal conduits for rainwater collection, concentration, and distribution. The distance-to-water map (Fig. 8b) was developed from wadi networks extracted using high-resolution ALOS PALSAR elevation data, representing the spatial proximity to these vital hydrological features.

The regional lithological framework is dominated by igneous and metamorphic formations with inherently low infiltration capacities, which further amplifies the importance of wadies as primary zones for both surface water accumulation and groundwater recharge. During rainfall events, these drainage channels efficiently capture and concentrate runoff, promoting infiltration into underlying permeable alluvial sediments. Consequently, areas situated in closer proximity to wadis are not only more favorable for accessing transient surface water but also exhibit a higher potential for shallow groundwater occurrence.

Within the spatial modeling framework, such zones are assigned higher suitability ranks for settlement, reflecting their hydrological advantages and strategic importance for ancient human occupation. This relationship highlights the principal role of wadi proximity in shaping historical settlement distribution and sustaining settlements within the arid landscapes of Al-Madinah region.

## 7. Results and discussion

A comprehensive settlement suitability analysis was conducted using a weighted overlay approach within a Geographic Information System (GIS) framework. This analysis integrated multiple spatial datasets representing key topographic, hydrological, geological, and ecological factors that influence historical and potential human settlement patterns across Al-Madinah region. Each thematic layer was standardized through reclassification into five suitability classes and assigned a relative weight based on its importance, as determined by the Analytical Hierarchy Process (AHP).

Among the six parameters analyzed, distance to water, slope, and soil type were identified as the most significant factors, each receiving an equal weight of 25%. This reflects the critical dependence of the region on ephemeral watercourses for both surface runoff and groundwater recharge, balanced with the requirements for terrain accessibility and soil stability. Elevation was assigned with a weight of 13%, while lithology and drainage density were each assigned weights of 6%, acknowledging their essential, though secondary, roles in shaping habitable zones. The integration of these reclassified and weighted layers produced a composite settlement suitability map for the entire administrative extent of Al-Madinah. This spatial output delineates zones of high to low suitability, reflecting geomorphological, hydrological, and ecological gradients across the region (Table 2).

**Table 2:** Parameters, weights, and ranking classes used in settlement suitability analysis.

Parameter	Weight (%)	Classes	Rank
Distance to Water (Meter)	25	0–1,000	5
		1,001–3,000	4
		3,001–6,000	3
		6,001–10,000	2
		10,000.1 - 49,651.6	1
Slope (Degree)	25	0.0–4.5	5
		4.6 - 12.8	4
		12.9 - 21.5	3
		21.6 - 31.5	2
		31.6 - 81.7	1
Soil	25	Calciorthids	5
		Torriorthents	4
		Torripsamments	3
		Salorthids	2
		Lava Flows	1
Elevation (Meter)	13	0.0 - 390.3	5
		390.4 - 724.8	4
		724.9 - 975.8	3
		975.9 − 1,208.2	2
		1,208.3 − 2,370	1
Drainage Density (Km/Km^2^)	6	0.0 - 0.019	1
		0.02 - 0.046	2
		0.047 - 0.073	3
		0.074 - 0.109	4
		0.11 - 0.181	5
Lithology	6	Harrat	1
		Igneous Rock	2
		Sedimentary Rock	5
		Metamorphic Rock	3

**Fig 9:**
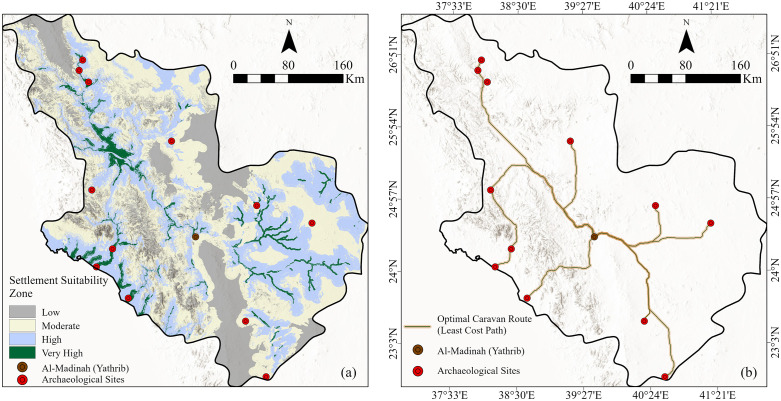
(a) Settlement suitability map of the Al-Madinah Region and (b) Least-cost path network from Yathrib to major archaeological sites. The spatial modeling and network analysis were conducted using ArcGIS Pro (Esri, Inc., 2023).

The resulting settlement suitability map (Fig 9a) categorizes the region into four suitability classes: low, moderate, high, and very high. According to area statistics (Table 3), the majority of the region falls within the moderate (44.13%) and high (32.01%) suitability classes. Low-suitability zones are predominantly located in high-elevation areas and rugged volcanic fields (Harrat), where steep terrain and inhospitable geological conditions limit permanent settlement due to restricted accessibility and scarce water resources.

**Table 3:** Area and percentage distribution of settlement suitability classes in Al-Madinah region.

Rank	Settlement suitability	Area (km2)	Percentage
1	Low	28,653.59	20.00
2	Moderate	63,205.33	44.13
3	High	45,846.41	32.01
4	Very High	5,529.02	3.86
Total		143,234.35	100.00

In contrast, areas classified as high and very high suitability are closely aligned with major wadi systems, which serve as the primary water sources in this arid environment. Given the prevalence of igneous and metamorphic bedrock, groundwater availability is generally constrained and localized, primarily accumulating within the alluvial beds of wadies. These fluvial corridors not only facilitate soil and vegetation development, essential for human settlements, but also reflect a historical continuity of settlement patterns dependent on surface runoff and episodic rainfall. The spatial correlation between very high suitability zones and wadi networks underscores the functional role of hydrological corridors in shaping both past and potential settlement dynamics.

To further assess movement and connectivity across this challenging terrain, a least-cost path analysis was conducted to reconstruct potential caravan routes toward Yathrib (Al-Madinah) (Fig 9b). The analysis utilizes a dedicated cost surface calibrated to represent movement friction across the landscape. This friction surface prioritizes low-energy corridors, specifically identifying wadi systems as optimal pathways while accounting for the high resistance of volcanic fields (Harrat). The model identified the most efficient routes connecting Yathrib to major archaeological centers, with calculated distances such as 189.37 km to Khaybar, 198.90 km to Al-Rabadha, and 352.30 km to Hegra.

These modeled paths exhibit strong alignment with known archaeological locations, many of which are strategically situated within the computed optimal corridors. This correspondence validates the model’s predictive capacity, demonstrating that terrain ruggedness and hydrological accessibility were the decisive guiding ancient human movement and caravan logistics. The resulting reconstruction provides a robust representation of historical connectivity and trade dynamics across the northwestern Arabian Peninsula.

**Fig 10:**
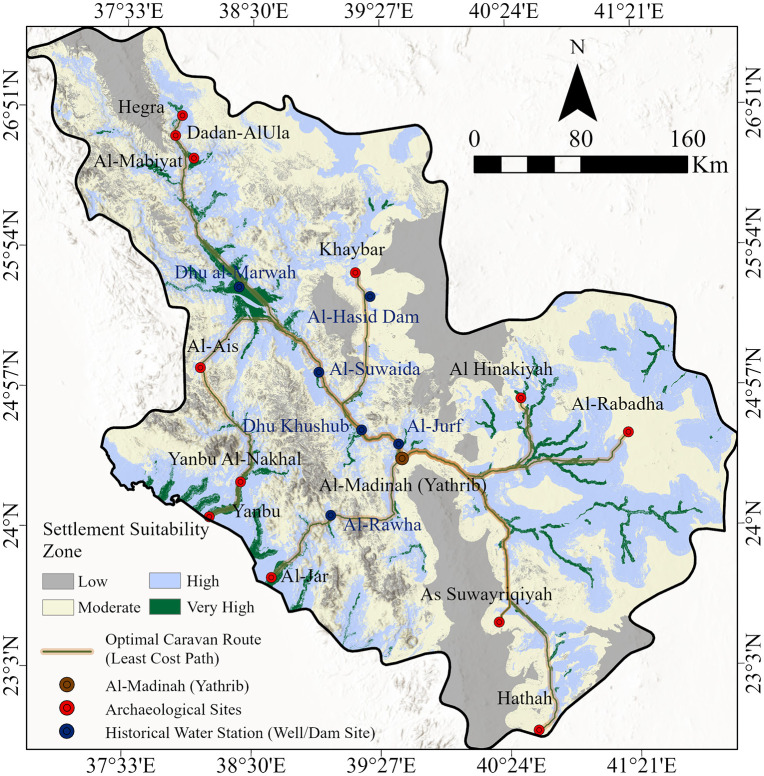
Final least-cost path map showing the alignment of optimal caravan routes with archaeological sites and historical water stations (wells and dams). Cartographic synthesis and path modeling were performed in May 2025 using ArcGIS Pro (Esri, Inc., 2023). Historical water source locations were identified, (modified after [2]).

The model’s reliability is quantitatively reinforced by the spatial proximity between the least-costly reconstructed corridors and historical water stations (Fig 10). A proximity analysis using the Near tool was conducted to measure the displacement between the modeled LCP and key archaeological stations along the primary Yathrib-Hegra trade route (Table 4). The results demonstrate a high degree of spatial accuracy, with a mean proximity error of 2.17 km across the entire corridor. This alignment is particularly precise at major historical centers such as Dhu Khushub (136.92 m) and Dadan-AlUla (158.15 m), where the modeled path shows near-perfect correspondence with documented sites.

**Table 4:** Quantitative validation of Least Cost Path (LCP) against historical trade stations (Yathrib – Hegra Route).

Station name	Route	Near distance (m)	Near distance (km)
Al-Jurf	Yathrib – Hegra	424.26	0.42
Dhu Khushub		136.92	0.14
Al-Suwaida		1006.12	1.01
Dhu al-Marwah		4962.89	4.96
Al-Mabiyat		6344.89	6.34
Dadan-AlUla		158.15	0.158
Mean		2172.21	2.17

While certain locations like Al-Mabiyat exhibit a localized deviation (6.34 km), such variances are expected and likely reflect tactical site-selection criteria, such as defensive requirements or localized topographical advantages, which transcend purely friction-based movement. Despite these minor anomalies, the strategic co-location of the LCP with water-endowed stations, including Al-Jurf, Al-Suwaida, and Dhu al-Marwah, confirms the model’s predictive capacity. These stations provided the essential hydrological infrastructure, such as wells and dams, necessary for sustaining long-distance caravans within the arid Hijaz highlands.

The spatial coherence among high settlement suitability areas, least-cost corridors, and historical water stations confirms that the hydrological accessibility and terrain friction were the primary determinants of ancient mobility. By transitioning from qualitative descriptions to specific distance metrics, this framework provides a reproducible validation that links environmental affordances with the historical spatial organization of the Al-Madinah region.

## 8. Conclusion

This study employed a Multi-Criteria Spatial Analysis (MCSA) framework integrating Geo-graphic Information Systems (GIS), the Analytic Hierarchy Process (AHP), and Least-Cost Path (LCP) modeling to evaluate historical settlement suitability and reconstruct optimal caravan routes converging on Yathrib (Al-Madinah). By addressing a critical gap in the spatial modeling of ancient human activity within arid environments, the research provides a methodologically robust framework for interpreting historical mobility, settlement patterns, and land use across the Al-Madinah Region.

Six key environmental and topographic parameters were analyzed: lithology, elevation, slope, drainage density, distance to water, and soil characteristics. Through an iterative AHP calibration that achieved a perfect consistency ratio (CR = 0.01), distance to water, slope, and soil suitability emerged as the primary determinants, each assigned a balanced weight of 25%. This approach corrected previous hydrological biases and demonstrated that while a significant portion of the region exhibits moderate settlement potential (44.13%), high-suitability zones (32.01%) are strategically concentrated along major wadi networks, highlighting the enduring role of these corridors in sustaining human settlements within predominantly impermeable geological formations.

Building upon this environmental framework, a dedicated cost surface was developed independently to simulate plausible caravan routes radiating from Yathrib toward major archaeological sites. The modeled paths exhibit a high degree of spatial accuracy, verified through a quantitative proximity analysis that yielded a mean error of only 2.17 km across the primary corridors. The near-perfect alignment with key historical stations, such as Dhu Khushub and Dadan-AlUla, underscores the spatial logic underpinning ancient mobility. Importantly, the convergence of these routes with verified historical water infrastructure—wells, dams, and catchments—further validates the model and highlights the critical interdependence between terrain friction and hydrological accessibility.

Overall, the study demonstrates that topography, water availability, and pedological factors jointly governed the spatial distribution of settlements and trade corridors in Al-Madinah region. This integrated GIS-based approach provides a replicable and quantitatively validated methodology for exploring ancient settlement connectivity in arid landscapes, offering valuable insights for both archaeological research and heritage management.

## References

[1] Al-Ghabban AI, André-Salvini B, Demange F, Juvin C, Cotty M. Roads of Arabia: archaeology and history of the kingdom of Saudi Arabia. Paris, France: Musée Du Louvre. 2010.

[2] Salama A. Urban centers along the incense route from southern Arabia to the port of Gaza: a study in historical geography. Scient J Faculty of Arts, Tanta Univ. 2024;55:2007–107.

[3] Al-Sharif A. Makkah and Madinah in the Jahiliyyah and the Era of the Prophet, 1st ed. Cairo, Egypt: Dar Al-Fikr Al-Arabi; 2003.

[4] Al-Naeem N. The economic situation in the Arabian Peninsula from the 3rd century BCE to the 3rd century CE. 1st ed. Riyadh, Saudi Arabia: Dar Al-Shawaf for Publishing and Distribution; 1992.

[5] Yao Y, Wang X, Luo L, Wan H, Ren H. An overview of GIS-RS applications for archaeological and cultural heritage under the DBAR-heritage mission. Remote Sensing. 2023;15(24):5766. doi: 10.3390/rs15245766

[6] Wiseman J, El-Baz F. Remote sensing in archaeology. Berlin/Heidelberg, Germany: Springer; 2007.

[7] Casana J, Cothren J, Kalayci T. Swords into ploughshares: archaeological applications of CORONA Satellite Imagery in the Near East. IA. 2012;(32). doi: 10.11141/ia.32.2

[8] Hritz C. Contributions of GIS and satellite-based remote sensing to landscape archaeology in the Middle East. J Archaeol Res. 2014;22:229–76. doi: 10.1007/s10814-013-9072-2

[9] Blom RG, Crippen R, Elachi C, Clapp N, Hedges GR, Zarins J. Southern Arabian desert trade routes, frankincense, myrrh, and the ubar legend. In: Wiseman J, El-Baz F, editors. Remote sensing in archaeology. New York, NY, USA: Springer; 2006. 71–87. doi: 10.1007/0-387-44455-6_3

[10] White DA. The basics of least cost analysis for archaeological applications. Adv archaeol pract. 2015;3(4):407–14. doi: 10.7183/2326-3768.3.4.407

[11] Carroll F, Carroll E. Budget travel in the Mediterranean: a methodology for reconstructing ancient journeys through least cost networks. J Comp Appl Archaeol. 2022;5(1):35–56. doi: 10.5334/jcaa.88

[12] Verhagen P, Nuninger L, Groenhuijzen MR. Modelling of pathways and movement networks in archaeology: An overview of current approaches. In: Verhagen P, Joyce J, Groenhuijzen MR, editors. Finding the limits of the limes: Modelling demography, economy and transport on the edge of the Roman Empire. Cham, Switzerland: Springer; 2019. 217–49.

[13] Frachetti MD, Smith CE, Traub CM, Williams T. Nomadic ecology shaped the highland geography of Asia’s Silk Roads. Nature. 2017;543(7644):193–8. doi: 10.1038/nature21696 28277506

[14] Städtler A. Evaluating ancient Arabia’s trade routes with least-cost-path analysis. In: The ancient world goes digital. Case studies on archaeology, texts, online publishing, digital archiving and preservation. 2023. 21–45.

[15] Ghadban Y. The city of Yathrib before Islam. 1st ed. Amman, Jordan: Dar Al-Bashir Publishing; 1993.

[16] Herzog I. Least-cost paths – some methodological issues. Internet Archaeol. 2014;(36). doi: 10.11141/ia.36.5

[17] El-Sorogy AS, Alharbi T, Al-Kahtany K, Rikan N, Salem Y. Identification and validation of groundwater potential zones in Al-Madinah Al-Munawarah, western Saudi Arabia using remote sensing and GIS techniques. Water. 2024;16(3421).

[18] Al-Mukhlafi AA. Was Yathrib a Kingdom? A historical study on the emergence and development of the city before Islam. J Historical Stud. 2019;2.

[19] Brown GF, Schmidt DL, Huffman Jr AC. Geology of the Arabian Peninsula; shield area of western Saudi Arabia. US Geological Survey. 1989.

[20] Metwaly M, Abdalla F, Taha AI. Hydrogeophysical study of sub-basaltic alluvial aquifer in the southern part of Al-Madinah Al-Munawarah, Saudi Arabia. Sustainability. 2021;13(17):9841. doi: 10.3390/su13179841

[21] Alsuhaibani A, Metwaly M. Integrated results of aerial image, ground magnetics and excavation for settlement assessment at Dadan site, Al‐’Ula area, Saudi Arabia. Archaeol Prospect. 2020;27(3):263–74. doi: 10.1002/arp.1770

[22] Alaska Satellite Facility. ALOS PALSAR 12.5m digital elevation data. Accessed 2025 May 6. https://search.asf.alaska.edu/#/?dataset=ALOS

[23] Pellaton C. Geologic map of the al madinah quadrangle. Kingdom of Saudi Arabia Deputy Ministry for Mineral Resources; 1981.

[24] Saaty TL. The analytic hierarchy process. New York, NY, USA: Mcgraw Hill; 1980.

[25] ArcGIS Pro. Redlands, CA, USA: Environmental Systems Research Institute; 2023.

[26] Yeh H-F, Cheng Y-S, Lin H-I, Lee C-H. Mapping groundwater recharge potential zone using a GIS approach in Hualian River, Taiwan. Sustain Environ Res. 2016;26(1):33–43. doi: 10.1016/j.serj.2015.09.005

